# Proposition of Two Subtypes of Patients at Risk of Suicide: Pain Hypersensitive Vs. Dissociative

**DOI:** 10.1007/s11920-025-01600-0

**Published:** 2025-03-17

**Authors:** Francesca Bianco, Philippe Courtet, Emilie Olié, Jorge López-Castroman, Fabio Madeddu, Raffaella Calati

**Affiliations:** 1 Department of Psychology, University of Milan-Bicocca, Milan, Italy; 2 Institut de Génomique Fonctionnelle, University of Montpellier, CNRS-INSERM, Montpellier, France; 3 Department of Emergency Psychiatry and Acute Care, Lapeyronie Hospital, CHU Montpellier, Montpellier, France; 4 FondaMental Foundation, Créteil, France; 5 Department of Adult Psychiatry, Nimes University Hospital, Nimes, France; 6 Center of Biomedical Network Research on Mental Health (CIBERSAM), Madrid, Spain; 7 Department of Signal Theory and Communication, Universidad Carlos III, Madrid, Spain

**Keywords:** Suicide, Phenotypes, Dissociation, Inflammation, Interoception, Pain

## Abstract

**Purpose of Review:**

The pain-suicide relationship is one of the most debated in recent literature, but theories and clinical evidence have often reached contrasting conclusions. Through a critical overview of theoretical, meta-analytical and empirical contributions, we aimed at advancing the conversation on the pain-suicide relationship by integrating research on related concepts, specifically inflammation and dissociation, and their effects on interoceptive processes and pain perception.

**Recent Findings:**

Ideation-to-action theories consider increased pain tolerance a key risk factor for the transition from suicidal ideation to attempt. However, several meta-analytical findings suggest that suicidal thoughts and behaviors are associated with inflammation-induced pain sensitization. On the one hand, inflammation contributes to the development and maintenance of chronic pain conditions and mood disorders, and is associated with interoceptive hypervigilance and pain hypersensitivity. Moreover, a trait of increased pain tolerance does not seem to distinguish the individuals attempting suicide among those living with suicidal thoughts. On the other, temporary hypoalgesia is often activated by dissociative experiences. Highly dissociative individuals can indeed be exposed to frequent disintegration of interoceptive processes and transitory hyposensitivity to pain. In light of this, two different patterns of responses to stress (i.e. inflammation vs. dissociation) may characterize different kinds of patients at risk of suicide, associated with specific patterns of interoceptive functioning, pain sensitivity and possibly suicidal ideation. This proposition is partially supported by neuroimaging studies on post-traumatic stress disorder and psychodynamic perspectives on neurodevelopment, as well as alternative clustering models of suicidal behavior.

**Summary:**

Theoretical, meta-analytical and neurobiological evidence highlight two opposite directions in the pain-suicide relationship: hyper- vs. hyposensitivity. Such contrasts may be explained by the existence of two tendencies in stress-response, namely inflammation and dissociation, defining two different subtypes of patients at risk of suicide. We thus propose the existence of a *hypersensitive subtype*, defined by underlying neuroinflammatory processes, increased vulnerability to chronic pain and mood disorders, interoceptive hypervigilance, pain hypersensitivity and potentially more persistent suicidal ideation. We further hypothesize a *dissociative subtype*, characterized by greater trait dissociation, vulnerability to depersonalization and derealization, frequent disintegration of interoceptive processes, transient pain hyposensitivity and abrupt peaks in suicidal ideation.

## Introduction

Suicide is one of the leading causes of death worldwide [1]. With a phenomenology comprising a wide range of thoughts and behaviors, suicide risk is often conceptualized along a continuum of increasing degrees of severity [2]. While the global estimates indicate that more than 9% of individuals experience suicidal ideation (SI) during their lifetime, only 29% of them enact a suicide attempt (SA) [3]. For this reason, recent models often refer to an *ideation-to-action* framework, studying the contributors to the progression from suicidal thoughts to their enactment, in order to improve screening, assessment, and prevention of suicide risk [4]. Substantial evidence suggests that pain, physical and psychological, is a key element in suicide risk [5–17]. Ideation-to-action theories all identify blunted pain perception as a primary factor in the transition from SI to SA, especially for lethal or near-lethal SA [18–20]. Pain-related aspects are some of the most debated topics in recent suicide literature. Although abundant, research on the connection between pain and suicide has at times reached contrasting conclusions. In particular, the role of pain sensitivity in facilitating the transition from SI to SA still warrants further investigation. This work aims to highlight key evidence on the connection between pain and suicide while proposing a new conceptual model.

### Pain and Suicide

A lot has been written about the link between pain and suicide. Only on PubMed, no less than 14 systematic-reviews and meta-analyses have been published over the course of the last 10 years containing the keywords ‘pain’ and ‘suicide’ in their title, whose findings will be detailed in the following paragraphs. Most of them revolve around two main aspects that have been hypothesized to pose a higher suicide risk, namely physical pain [5–12] and psychological pain [13–17]. Only one record [21] focuses on physical pain sensitivity among people with a history of SA.

The records concerning physical pain indicate that individuals living with any kind of physical pain report higher rates of death wishes, SI, suicidal planning, SA and death by suicide, as well as non-suicidal self-injury (NSSI) [5–12]. Such relationships are not limited to the adult population but were also found in adolescent clinical and community samples [6, 8, 12]. Among the factors related to higher rates of suicidal behavior in chronic pain patients, comorbid psychiatric conditions were often mentioned, especially depressive disorders [6, 9–11] but also anxiety disorders [10], post-traumatic stress disorder (PTSD) and borderline personality disorder (BPD) [9].

According to Shneidman’s model of suicide [22], psychache, or psychological pain, which arises when vital psychological needs are unmet, is considered the main psychological risk factor for suicide vulnerability, when deemed unbearable by the subject. The records focusing on this kind of pain highlighted that higher levels of psychological pain were associated with higher rates of SI and SA in mood disorder patients, other psychiatric patients and nonclinical samples [13, 14, 17]. The association remained significant even when controlling for depression [17] and psychological pain seemed to be a stronger predictor of SI than depression [13]. A systematic review of magnetic resonance imaging (MRI) studies found alterations among adolescents with SI and SA histories in an emotional pain circuit, composed of cerebellum, amygdala and hippocampus. However, alterations in what they defined as a ‘social disconnect’ circuit, composed of lateral orbitofrontal cortex, temporal gyrus and their connections, were specific to those with a history of SA [15]. Moreover, hypofunctioning of cannabinoid receptors connected to the modulation of neuropathic pain, which might also play a role in modulating mental pain, distinguished patients with SA history from those without [16].

One meta-analysis [21] examining physical pain thresholds and tolerance in people with a history of SA challenged the ideation-to-action frameworks’ hypothesis that increased pain tolerance heightened the risk of transitioning from suicidal thoughts to SA [18–20]. Indeed, while pain tolerance was higher among people with a history of SA, as compared to nonclinical controls, neither pain threshold nor tolerance reliably differentiated people attempting suicide from other psychiatric patients. Altered pain perception seemed to characterize overall psychiatric vulnerability.

### Pain Hypersensitivity and Neuroinflammation

Since both physical and psychological pain appear to play a pivotal role in suicide-related thoughts and behavior, it is worth introducing a concept consistently linked to both: neuroinflammation. Inflammation is relayed to the brain by the immune system through interoceptive visceral afferent pathways, as well as humoral and cellular interoceptive pathways. Inflammatory processes are also mediated by top-down projections from the brain that modulate autonomic sympathetic and parasympathetic responses [23, 24]. Acute inflammation is an adaptive response to physical or emotional stress that serves to reprioritize physiological responses to address potential threats. However, chronic inflammation causes long-lasting biochemical changes that contribute to the development and maintenance of chronic pain conditions [25–29] and psychiatric disorders, particularly depressive disorders [23, 26, 30].

For example, prolonged neuroinflammation may lead to persistent central sensitization, a phenomenon in which maladaptive neuroplasticity induces lasting hyperexcitability of central pain pathways, resulting in amplified pain processing and pain hypersensitivity [31–33]. Central and peripheral inflammation play a role in the pathogenesis of chronic pain [34] and patients with chronic pain conditions often possess hypersensitivity to physical pain, expressed by lower pain thresholds compared to healthy controls [35]. Indeed, neuroinflammatory processes lead to heightened processing of aversive interoceptive stimuli and increased interoceptive hypervigilance, reflected by abnormal activation in the posterior insula, dorsolateral prefrontal cortex (PFC), anterior cingulate cortex (ACC) and somatosensory cortex [32, 36]. Moreover, neuroinflammation tends to inhibit fear extinction processes, thus strengthening the neural signature of interoceptive fear memory traces [32, 36, 37]. Interoception, defined as the sensing of the physiological condition of the body [38–40], intertwines with many metacognitive dimensions, such as cognitive appraisal and interpretation, affective evaluation, as well as the degree of trust and attention given to interoceptive sensations [41]. Sustained neuroinflammation also affects these components facilitating stress-sensitization processes and thus altering emotional processing and affect regulation [42]. Consistently, interoceptive signaling of inflammation appears to be involved in the etiology of mood disorders [30] and metacognitive components of interoception are thought to be implicated in the pathogenesis of some anxiety disorders, such as panic disorder [43]. Additionally, a possible adverse consequence of inflammation-induced interoceptive hypervigilance is pain catastrophizing, one of the most important correlates of pain chronicity and disability [44]. During resting-state fMRI, patients living with chronic low-back pain displayed abnormal connectivity between the amygdala and the ‘central executive network’, which may be associated with increased pain catastrophizing and rumination [45]. Finally, since the neural networks involved in physical and psychological pain processing partially overlap [46–49], neuroinflammation could also induce hypersensitivity to psychological pain [30, 50].

Not surprisingly, consistent evidence also suggests an involvement of peripheral and neuroinflammatory processes in suicidal behavior [50–53]. Increased pain sensitivity has indeed been observed among older adults with SI and SA histories, who also recurred more frequently to opioid analgesics and reported a greater impact of pain on their quality of life [54, 55]. Moreover, interoceptive dysfunctions, especially in cognitive and emotional appraisal of bodily sensations, were associated with suicide-related outcomes [56, 57]. More specifically, lower trust in bodily sensations was connected to both SI and SA among the general population, while those with a history of SI seemed to be particularly characterized by lower capacity to regulate worry about unpleasant bodily sensations [58]. However, results were different using different interoceptive measures [59].

Such findings align with the work of Elman and colleagues [60], who highlighted the role of altered anti-reward brain networks in chronic pain conditions and suicidal behavior, similarly to what was observed in addictive behaviors. Prolonged pain or stress may indeed produce desensitization of reward and sensitization of anti-reward networks, alongside pain-stress cross-sensitization. This translates into aberrant learning processes, increasing incentive salience attribution to pain-related stimuli and enhancing responsivity to stress and negative emotional states. Similarly, a suicide-stress cross-sensitization is also theorized based on evidence of strong links between life adversities and suicide-related behaviors. Consistently, increased frequency and lethality of SA observed in some patients could in part be explained by aberrant salience attributed to suicide-related content. Similarly to addictive behaviors, pain-stress and suicide-stress cross-sensitization may narrow the behavioral repertoire and facilitate habit-based, pseudo-compulsive self-injurious and suicidal behaviors.

Considering all this evidence, contrary to what is hypothesized by ideation-to-action theories, at least some individuals at risk of suicide, such as those with depression and chronic pain, may possess enhanced pain sensitivity. These patients may belong to a *hypersensitive subtype* of individuals at risk of suicide, characterized by inflammation-induced central sensitization, impaired interoceptive pain, and fear extinction processes and sensitized anti-reward brain networks. Such individuals may struggle to shift their attention away from psychological and physical pain, as well as suicide-related content, leading to pain catastrophizing, hopelessness and potentially more persistent SI. However, while we suggest that some at-risk patients may belong to this hypersensitive subtype, not all individuals at risk of attempting suicide will necessarily exhibit this exact pattern.

### Pain Hyposensitivity and Dissociation

Despite strong evidence linking suicide risk to heightened pain sensitivity, most recent theories of suicide emphasized increased pain tolerance as a risk factor in the transition from suicidal ideation to action [18–20]. This apparent contradiction between empirical findings and theoretical models warrants a closer examination of the origins of the increased pain tolerance hypothesis and a reconsideration of current conceptualizations.

Pain hyposensitivity as a risk factor for suicide was first proposed by Orbach and colleagues, who viewed it as part of a broader indifference toward bodily sensations, stemming from a dissociative disposition, which was thought to facilitate self-aggression [61]. Consistent with this theory, they found that individuals hospitalized after a suicide attempt exhibited higher pain thresholds and tolerance [62–64], lower interoceptive abilities [65] and higher levels of dissociation [66] compared to both psychiatric inpatients without a history of SAs and non-clinical controls. Later, Joiner’s Interpersonal-Psychological Theory of Suicide (IPTS) [20, 67] introduced the concept of an acquired capability for suicide (CS), suggesting that suicidal desire arises from frustrated belongingness and perceived burdensomeness. Even so, the instinctual fear of pain and death and the drive to self-preservation prevent most people with suicidal thoughts from attempting suicide. However, repeated exposure to painful or fear-inducing events (e.g. combat, violence, road accidents and physical injuries or illnesses) was hypothesized to increase pain tolerance and fearlessness about death, desensitizing the individual to self-preservation instincts and posing the foundation for the acquired CS. Later, the Three-Step Theory [18] proposed that the CS arises from three sources: acquired, dispositional and practical contributors. While acquired contributors align with IPTS (e.g., repeated exposure to pain or fear), practical contributors involve the access to and knowledge of lethal means. Dispositional contributors refer to temperamental, personality and genetic dimensions, such as low harm-avoidance and low pain sensitivity, which are deemed responsible for a diminished aversion to self-inflicted pain, injury, or death. Across these models, pain hyposensitivity has been conceptualized as a relatively stable trait-like characteristic, whether innate (dispositional) or developed through experience (acquired). However, recent empirical findings challenge this view, since they indicate that diminished pain perception, as a trait, does not reliably distinguish individuals who attempted suicide among those with SI history [21, 68].

Instead, temporary states of diminished pain perception, potentially induced by dissociative responses to acute psychological stress, may play a more crucial role. For example, Risch and colleagues noted that pain sensitivity in suicide attempters is typically assessed as a trait; therefore, temporary states of pain hyposensitivity immediately preceding the attempt might have gone undetected. Consistently, Cáceda and colleagues [69] found that patients who had recently attempted suicide exhibited significantly higher pain thresholds than those with only SI, but their thresholds decreased in a follow-up assessment after 3–5 days, becoming comparable to the controls. This suggests that pain sensitivity fluctuates in time; therefore, individuals who attempt suicide may possess comparable pain sensitivity to those with suicidal thoughts, but they may encounter acute decreases in pain perception right before the SA, as a result of entering a dissociative state. This aligns with evidence indicating that experimentally-induced dissociation produces reduced pain perception across various populations and experimental paradigms [70–72].

Consistently with Orbach’s hypotheses, a dissociative tendency may in fact facilitate the transition from suicidal thoughts to behavior. Meta-analytic and original studies have indeed found higher rates of SA and NSSI in patients with Dissociative Disorders (DDs) and higher dissociative traits (measured with the Dissociative Experiences Scale, DES) in psychiatric patients with SA or NSSI versus psychiatric patients without them [73–75]. Additionally, a recent systematic review found alterations in interoception along the suicidality continuum [56]. In particular, a greater tendency to distract from uncomfortable bodily sensations was associated with a history of SA [58]. Dissociative symptoms are also often associated with interoceptive abnormalities. For example, patients diagnosed with DDs exhibited lower objective interoceptive accuracy compared to healthy controls in heart-beat detection tasks [76]. The most significant associations between dissociation and interoceptive processes, however, seem to involve alterations in conscious processing of pain perception and integration of affective and motivational aspects of pain. Indeed, Ducasse and colleagues [77] revealed increased tolerance to acute and self-inflicted pain among patients with BPD, who often experience dissociation and engage in SA or NSSI. Based on neuroimaging evidence [78–80], their hypoalgesia did not seem to depend on impaired sensory discrimination, derived from altered transmission of nociceptive input to the somatosensory and cingulate cortices. Instead, the authors suggested an involvement of an exaggerated intracortical pain control, associated with attentional shifts, altered integration of cognitive and emotional processes, and abnormal appraisal of cognitive, motivational and affective aspects of pain.

Not surprisingly, dissociative processes seem to rely on the activity of key brain regions also involved in interoceptive and pain-modulation processes. Interoceptive information is processed in the insular cortex (IC), where it proceeds in a posterior-to-anterior progression toward its integration with a conscious experience of self [81]. More specifically, the posterior portions of the IC provide primary representations of bodily sensations, while the anterior IC is involved in subjective and emotional appraisal of painful stimuli. In particular, the anterior IC integrates sensory information through the interaction with other regions, such as the ACC and PFC [81–85], which modulate pain and affect through descending pathways to the amygdala and periaqueductal gray [86–90]. Dissociative responses during emotionally triggering situations seem to be associated with hyperactivation of ACC and PFC and hyperinhibition of amygdala responses [91, 92]. Additionally, a recent meta-analysis examined the neurofunctional correlates of dissociative symptoms across different diagnoses, highlighting altered activity in some of these regions. In particular, decreased activation of parahippocampal gyrus, insula and amygdala and enhanced response of middle frontal gyrus, cingulate cortex and hippocampus were found to be neurobiological markers across the whole dissociative spectrum [93]. Most interestingly, preliminary findings from a study using resting-state fMRI identified altered activity in the IC, cingulate cortices and PFC as possible biomarkers of the CS, supporting a relationship between the CS and abnormal cortical modulation of pain [94]. Lastly, neurobiological evidence suggests that the analgesic effect of some defensive responses to stress, such as stress-induced dissociation, could in part rely on the contribution of the endogenous opioid system. More specifically, alterations in both the mu- and kappa-opioid systems seem to be involved, respectively, in the analgesic effects of stress and in experiences of disrupted consciousness [95].

Pain hyposensitivity as a trait may not be as central as ideation-to-action models suggest, at least not as a distal risk factor on its own. Instead, transient reductions in pain perception, driven by dissociative responses, could act as precipitating factors that facilitate the progression from suicidal thoughts to action. For instance, Courtet & Guillaume [50] theorized that individuals with a greater tendency toward dissociation may frequently experience states of bodily disconnection, associated with blunted pain perception. Indeed, we propose that greater trait dissociation could be a stronger dispositional contributor to the CS than increased pain tolerance. We further propose that at least a subgroup of people at risk of suicide may belong to a *dissociative subtype*, characterized by greater trait dissociation, which predisposes them to frequent disruptions of interoceptive processing, particularly its affective and metacognitive components. This subgroup may be especially prone, perhaps in response to internal or external stressors, to entering dissociative states. During these states bodily sensations, including pain, are substantially altered by cognitive and attentional processes and/or relatively disconnected from emotional experience, resulting in acute decreases in pain perception.

### Opposite Patterns of Stress Response

If our proposition of two subtypes of patients at risk of suicide holds true, it would be coherent with the line of evidence that led to the addition of a dissociative subtype of PTSD in the Diagnostic and Statistical Manual of Mental Disorders, Fifth Edition (DSM-5) [96], reaffirmed in its latest revision (DSM-5-TR) [97]. Using fMRI in a script-driven imagery paradigm, Lanius and colleagues found that most PTSD patients reported re-experiencing and hyperarousal responses during traumatic memory recall, accompanied by increased heart rate. In contrast, a subgroup of patients reported dissociative states of depersonalization/derealization and displayed no significant changes in heart rate. The two groups also revealed opposite neurofunctional activation patterns in response to trauma-related stimuli. Patients with re-experiencing symptoms exhibited hyperactivation in the right anterior insula and amygdala, accompanied by abnormally reduced activation of the ventromedial PFC and the ACC, indicating impaired cortical regulation of emotional and arousal responses. Conversely, the dissociative subgroup exhibited hyperactivation in the ventromedial PFC and the ACC, alongside inhibition of the IC and limbic regions, including the amygdala, revealing a pattern of overmodulation of emotional and arousal responses to trauma-related content [91, 98]. Such findings support the theories proposed by Perry [99] and then Bremner [100], which suggest the existence of two opposite pathways of acute traumatic response: hyperarousal and dissociation.

Neuropsychoanalytic contributions have also identified the same two patterns in the context of early relational trauma and the neurobiological responses activated during disturbed child-caregiver interactions [101–103]. According to this psychodynamic perspective on neurodevelopment, the symptoms and traits emerging following trauma reflect the predominant response pattern at the time of and immediately after the traumatic event. A primarily dissociative response may strengthen the neurobiological circuits underlying dissociative processes, predisposing to the development of dissociative traits and symptoms. Conversely, if a hyperarousal response prevails, the associated neurobiological system becomes sensitized, making the individual more prone to develop hypervigilance, increased emotional reactivity and maladaptive emotion regulation strategies [99, 104, 105]. In line with such findings, we propose that similar patterns may be found among individuals that are at risk of attempting suicide, which may possess specific tendencies toward dissociation or hyperarousal in response to intense, emotionally triggering situations.

It is also possible that our proposed model is best suited for individuals at suicide risk who have experienced psychological trauma, while other at-risk individuals may not fit within this framework. Indeed, early life adversities and traumatic experiences are strongly associated with an increased risk of developing chronic pain conditions and depression [7, 106, 107], as well as dissociative symptoms and traits [108–110]. Additionally, a fMRI/script-driven imagery paradigm has also been applied to studying the neurofunctional correlates of suicidal behavior, yielding intriguing findings [111]. In a sample of individuals with a recent history of SA, researchers observed decreased activation in the dorsolateral, anterior and medial PFC when participants recalled the mental pain associated with their SA. Compared to mental pain recalling, the mere recalling of the sequence of suicidal actions was linked to increased activation in the medial PFC, ACC and hippocampus, consistent with goal-directed behavior. Given the central role of the ACC and medial PFC in interoceptive awareness [112] and in the modulation of emotional responses [87], these findings are consistent with the idea that suicidal behavior is a state-dependent condition involving cortical inhibition of emotional reactions. Furthermore, the similarity between these findings and those obtained in neuroimaging studies using script-driven imagery with PTSD patients led Reisch and colleagues to suggest that suicidal crises may possess the quality of acute traumatic states. This idea could also explain the high levels of dissociative symptoms frequently reported by patients with a history of SA. However, the authors did not investigate whether stronger activation patterns in the aforementioned brain regions during the recollection of suicidal actions were associated with higher levels of dissociative symptoms or traits. Nonetheless, if we conceptualize suicidal crises as traumatic in nature, it would not be surprising to find neurophysiological response patterns in individuals with SA histories that resemble those observed in traumatized patients [91, 98]. Future neuroimaging studies using script-driven imagery with people at risk of suicide could further test the validity of this hypothesis. In conclusion, the theories and evidence discussed here predominantly focus on clinical populations. Future research should examine whether these two patterns of stress response also manifest in non-clinical individuals, both with and without SI.

### A Specification on the Term ‘Dissociation’

At this point, a glance at what can be meant by ‘*dissociation’* is worth taking. Dissociation is classically defined as a ‘*lack of normal integration of thoughts*,* feelings*,* and experiences into the stream of consciousness and memory’* [113]. Alternatively conceptualized as a defense mechanism, a cognitive style, a personality trait or a constellation of symptoms, dissociative phenomena encompass milder and less maladaptive manifestations, such as absorption, suggestibility, phantasy proneness and daydreaming [114–116], as well as more severe pathological manifestations and disorders [117, 118].

Among dissociative symptoms, a common distinction is the one between *psychoform* symptoms, such as depersonalization/derealization, amnesia or identity fragmentation, and symptoms that are *somatoform* in nature, such as conversion symptoms [119]. Moreover, both psychoform and somatoform dissociation can determine *negative symptoms*, such as detachment (i.e. depersonalization and derealization), emotional numbing and/or physiological irresponsiveness to normally provocative stimuli, as well as *positive symptoms*, such as intrusive, often trauma-related, re-experiencing states like flashbacks and ‘body-memories’ of traumatic pain [93, 120]. The existence of positive/re-experiencing symptoms of dissociation possibly highlights a conceptual imprecision in the distinction between a re-experiencing/hyperaroused vs. dissociative PTSD: from many points of view, trauma-related re-experiencing symptoms are indeed dissociative in nature.

Therefore, we specify for the sake of clarity that by trait dissociation, in the present work, we mostly refer to a greater tendency of experiencing dissociative states in everyday life, as assessed for example by the DES [113, 121]. The DES measures different dimensions but focuses primarily on experiences of absorption and negative symptoms of *detachment* (e.g., depersonalization and derealization) and *compartmentalization* (e.g., amnesia, fragmentation of identity) [121]. Among the general population, some individuals possess higher levels of trait dissociation as assessed by the DES. These highly dissociative individuals do not only dissociate more frequently in their day-to-day life, but they are also more prone to experience laboratory-induced dissociation across different dissociation-inducing paradigms [122].

### Proposition of Two Subtypes

**Fig. 1:**
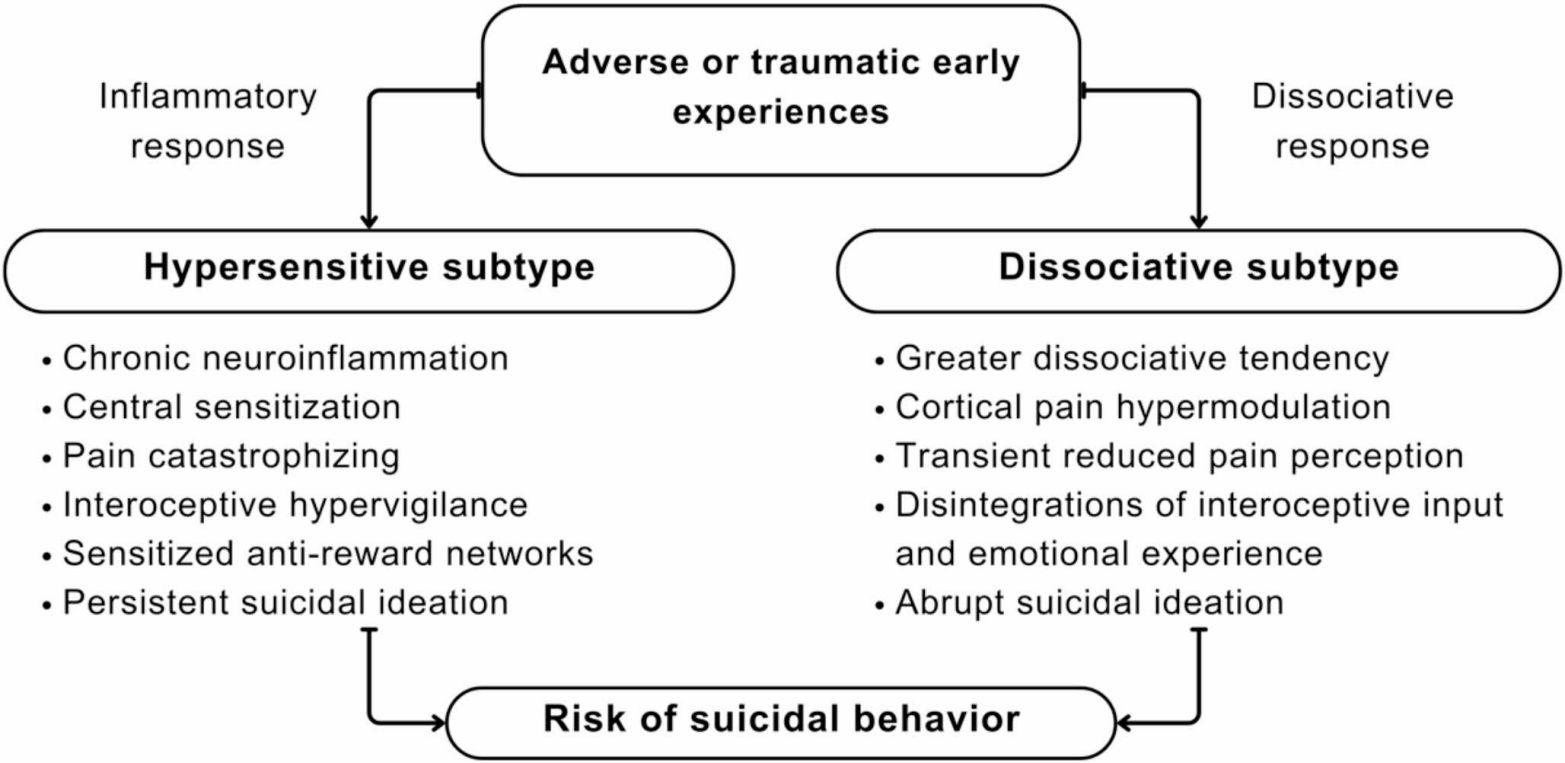
We hypothesize the existence of two subtypes of patients at increased risk of developing suicidal behaviors, defined by different stress-response patterns and specific profiles in terms of interoceptive functioning, pain processing and suicidal ideation. A *hypersensitive subtype* may have adapted to adverse experiences through a predominantly inflammatory response that may have chronicized over time into persistent central sensitization and interoceptive hypervigilance. Prolonged inflammatory processes also resulted in increased vulnerability to stress and impaired affect regulation, associated with greater risk of developing depressive symptoms, anxiety and chronic pain. Moreover, desensitization of reward and sensitization of anti-reward networks and difficulties in distracting from suicide and pain may associate with more persistent suicidal ideation. A *dissociative subtype* of patients may conversely have adapted to adverse experiences through predominantly dissociative responses, marked by excessive cortical modulation of pain and impaired integration of interoceptive input with conscious subjective emotional experience. This defense mechanism, solidified over time into a trait-like characteristic, facilitates the activation of dissociative states in the face of stress, accompanied by disruptions in interoceptive awareness, acute decreases in pain perception and more abrupt suicidal thoughts

With the present work, we propose the conceptualization of two different subtypes of patients at risk of suicide: a *hypersensitive* vs. a *dissociative subtype*, characterized by different patterns of pain perception and possibly specific ideation-to-action trajectories. In particular, we hypothesize that a trait of pain hypersensitivity, derived from inflammation-induced central sensitization, may predispose to the development of chronic pain condition, mood and anxiety disorders, and be accompanied by increased responsivity to stress, sensitized anti-reward networks, and difficulties in shifting attention away from pain and persistent suicidal thoughts. Conversely, a dissociative tendency appears to be associated with frequent disruptions in the integration between subjective metacognitive processes and interoceptive information, due to excessive cortical inhibition of pain, both physical and psychological, and resulting in acute decreases in pain perception and possibly more abrupt peaks in SI (Fig. 1).

Although we hypothesize the two subtypes to reflect relatively stable patterns of response to traumatic experiences and/or severe stress, it is important not to exclude the eventuality that one individual may present mixed characteristics of the two subtypes, or that a person may transition from one subtype to the other – in particular from the hypersensitive subtype towards the dissociative subtype – in response to significant stressful life events. An example of possible mixed characteristics can be found in a study by Ducasse and colleagues [77], where enhanced sensitivity to psychological pain was hypothesized to induce BPD patients to self-injure in the attempt of regulating negative emotions. The same patients, however, displayed blunted sensitivity toward acute self-induced physical pain. In another study, people with a history of SAs exhibited at the same time hypersensitivity to psychological pain, but hyposensitivity to bodily cues and physical dissociation, described as feelings of estrangement and detachment from the body [123]. These examples suggest a more complex relationship between psychological pain and dissociative responses. Indeed, detachment from bodily sensations, resulting in transient increased pain tolerance, can often be a defensive response against excessive psychological pain, activated in an attempt to restore a previous homeostatic state. It could be that, in certain vulnerable individuals, this may facilitate an aggression to the body. Coherently, a possible movement from hypersensitivity to dissociation has been suggested in a recent work by Courtet & Guillaume [50], where unbearable psychological pain was hypothesized to trigger dissociative processes in patients at risk of suicide. Although the relationship between pain and suicide has been widely studied, a lot of questions are yet to be answered, and the role of dissociative processes is one of them. From our perspective, transitioning from one subtype to the other is a possibility, but for now a merely speculative one, which will need empirical support in the future.

The notion of different profiles in patients at suicide risk is also not new to the field. Oquendo and colleagues [51, 124] have proposed the existence of two distinguished types of suicidal patients with specific trajectories towards suicidal behavior: (1) a stress-responsive type, rooted in a history of childhood trauma, characterized by elevated cortisol reactivity and more emotional/impulsive pathways towards SA; (2) and a non-stress-responsive type, connected to a history of depression with baseline dysfunctions in the serotonin system and a higher risk of engaging in meticulously planned SA. The two types also diverge in terms of patterns of SI, with type 1 being more prone to have abrupt peaks in SI in response to stressful life events, and type 2 exhibiting more persistent and severe SI. Furthermore, some studies based on hierarchical clustering also distinguished people with history of SA who engaged in impulsive, less planned and less lethal attempts, connected to weaker suicidal intent, and a minority of individuals engaging in well-planned and more lethal pathways toward attempting [125, 126].

Trying to align our hypotheses with previous models and considering the evidence from neurobiological studies on PTSD, our proposed dissociative subtype could also be seen as a subcategorization of Oquendo’s type 1. This subtype differs from the standard stress-responsive type by exhibiting a tendency to dissociate in response to trauma and inhibit emotional responses, rather than displaying enhanced emotional reactivity and impulsivity. As such, it is also plausible that stress-induced dissociative states may lead to less planned SA. Like emotional reactivity, a dissociative tendency can also be rooted in a history of childhood trauma, as DDs and symptoms have often been connected to childhood abuse [128–130]. Some authors also found that the presence of a DD fully mediated the effect of sexual abuse on the risk of multiple SAs, and they suggested that individuals who ‘adapt’ by developing DDs may meet a higher risk of engaging in self-harm and suicidal behaviors [74]. Conversely, our proposed hypersensitive subtype shares most characteristics with Oquendo’s non-stress-responsive type, such as its association with depression and a pattern of more persistent SI, potentially also connected with more planned SA. However, chronic pain conditions and depression in adulthood are also known to be linked to traumatic or adverse childhood experiences [7, 106, 107]. We additionally suppose the hypersensitive subtype to be somewhat sensitized to stress and more prone to anxiety. Even so, this sensitivity may not automatically translate into increased behavioral reactivity and impulsivity, but it may involve more cognitive components, such as excessive focus on pain and suicidal thoughts [7, 106, 107, 127]. Nonetheless, this comparison remains speculative, and whether the aforementioned subtypes overlap, even partially, to our proposed categorization is a question that requires further investigation.

### Antisuicidal Effect of Ketamine-Induced Dissociation?

Additional insights for our proposed theory may come from pharmacological studies on ketamine, known for its antisuicidal and antidepressive effects, but also for having dissociative and analgesic properties [131]. Strong evidence suggests that ketamine can quickly reduce SI, but fewer evidence exists about long-lasting anti-suicidal effects and prevention of suicidal behavior [131–133]. By disconnecting negative emotionality from conscious subjective experience, dissociative states may temporarily inhibit SI and depressive feelings. Highly dissociative individuals might naturally activate this short-term defense mechanism in stressful situations, but the same mechanism could also be pharmacologically induced by substances like ketamine. Moreover, the effects of dissociation may vary depending on the context. As enhancing synaptogenesis and neuroplasticity [134], a dissociative response to stressful events could enhance fearful memory consolidation, contributing to the development of later trauma-related symptoms [135]. Conversely, if experienced in the context of psychotherapy sessions, it may strengthen the beneficial effects of experiencing a trusting therapeutic relationship [134, 136].

## Conclusion

Our literature analysis reveals complex relationships between pain and suicide risk. Several key dimensions emerge from our investigation, including neuroinflammation, dissociation and distinct stress-response patterns. From our perspective, two distinct lines of evidence emerge linking suicide risk to both hyper- and hyposensitivity to pain, leading us to hypothesize the existence of two distinct subtypes of patients at risk of suicide. A *hypersensitive subtype* may be characterized by inflammation-induced central sensitization and increased vulnerability to chronic pain, anxiety and mood disorders. This kind of patients may display interoceptive hypervigilance and pain catastrophizing, because of impaired interoceptive pain and fear extinction processes. Moreover, due to sensitized anti-reward brain networks, they could encounter difficulties in shifting attention away from pain and suicide, potentially resulting in more persistent SI. A *dissociative subtype* may conversely be characterized by a greater tendency to activate dissociative responses in the face of stress, marked by disruptions in the integration between interoceptive input and subjective emotional experience due to excessive cortical modulation of pain, both physical and psychological. This kind of patients may encounter frequent states of depersonalization/derealization, in which they experience transient reductions in pain perception and potentially abrupt suicidal thoughts. This conceptualization aligns with the two stress-response patterns observed in PTSD [91, 98] and shares conceptual similarities with SI patterns described by Oquendo and colleagues [51, 124]. Our proposed framework primarily aims to address inconsistencies in the literature on pain and suicide. The hypothesis of a hypersensitive and a dissociative subtype of patients at risk of suicide may help integrate the valuable notions of neuroinflammation, dissociation and the metacognitive components of interoception into the discourse. Furthermore, the insights coming from trauma-related literature may help reconsider overall suicide-related phenomena as the result of the combination between specific tendencies of stress response and subsequent maladaptive adjustments in adult life. Further empirical investigation is necessary to verify the validity of our hypotheses. For example, clustering analyses focusing on dissociative traits, interoceptive awareness and pain perception among individuals with different patterns of suicide-related thoughts and behavior could enlighten our knowledge about potentially distinct ideation-to-action pathways. Moreover, it would be crucial to establish the degree to which a transition from one subtype to the other is possible, or even the likelihood to observe mixed characteristics or alternated modes of functioning in the same person. In order to inform screening and intervention practices on suicide risk, it would be particularly valuable to conduct experimental designs to investigate the directionality of these relationships. Furthermore, intensive longitudinal designs could capture the temporal fluctuations of these dimensions, in interaction with the ebb and flow of suicide-related and self-injurious thoughts and behaviors. If our proposed subtypes are confirmed by empirical evidence, they may sustain the development of more targeted and personalized interventions for different kinds of patients, possibly improving the treatments’ efficacy on patients at risk of suicide.

## Data Availability

No datasets were generated or analysed during the current study.

## Abbreviations

ACC: Anterior cingulate cortex
BPD: Borderline personality disorder
CS: Capability for suicide
DDs: Dissociative disorders
DES: Dissociative experiences scale
DW: Death wish
fMRI: functional magnetic resonance imaging
IC: Insular cortex
IPTS: Interpersonal psychological theory of suicide
NSSI: Non-suicidal self-injury
PFC: Prefrontal cortex
PTSD: Post-traumatic stress disorder
SA: Suicide attempt
SI: Suicidal ideation
SP: Suicidal planning

## References

[1] World Health Organization. Suicide Worldwide in 2019 Global Health Estimates. (2021).

[2] Posner K, et al. The Columbia–Suicide severity rating scale: initial validity and internal consistency findings from three multisite studies with adolescents and adults. Am J Psychiatry. 2011;168:1266–77. 22193671 10.1176/appi.ajp.2011.10111704 PMC3893686

[3] Nock MK, et al. Cross-National prevalence and risk factors for suicidal ideation, plans, and attempts. Br J Psychiatry. 2008;192:98–105. 18245022 10.1192/bjp.bp.107.040113 PMC2259024

[4] Klonsky ED, May AM. Differentiating suicide attempters from suicide ideators: A critical frontier for suicidology research. Suicide Life Threat Behav. 2014;44:1–5. 24313594 10.1111/sltb.12068

[5] Calati R, Laglaoui Bakhiyi C, Artero S, Ilgen M, Courtet P. The impact of physical pain on suicidal thoughts and behaviors: Meta-analyses. Journal of Psychiatric Research vol. 71 16–32 Preprint at 10.1016/j.jpsychires.2015.09.004 (2015). 10.1016/j.jpsychires.2015.09.004 26522868

[6] Hinze V et al. The relationship between pain and suicidal vulnerability in adolescence: a systematic review. The Lancet Child and Adolescent Health vol. 3 899–916 Preprint at 10.1016/S2352-4642(19)30267-6 (2019). 10.1016/S2352-4642(19)30267-6 PMC6842327 31606322

[7] Johnson BN, McKernan L. Co-Occurring Trauma and Non-Suicidal Self-Injury Among People With Chronic Pain: A Systematic Review. Current Pain and Headache Reports vol. 25 Preprint at 10.1007/s11916-021-00984-x (2021). 10.1007/s11916-021-00984-x PMC9332929 34766192

[8] Giakas A et al. Risks of suicide in migraine, non-migraine headache, back, and neck pain: a systematic review and meta-analysis. Frontiers in Neurology vol. 14 Preprint at 10.3389/fneur.2023.1160204 (2023). 10.3389/fneur.2023.1160204 PMC10157105 37153662

[9] Kwon CY, Lee B. Prevalence of suicidal behavior in patients with chronic pain: a systematic review and meta-analysis of observational studies. Frontiers in Psychology vol. 14 Preprint at 10.3389/fpsyg.2023.1217299 (2023). 10.3389/fpsyg.2023.1217299 PMC10576560 37842717

[10] Huang S et al. Predictive Modeling for Suicide-Related Outcomes and Risk Factors among Patients with Pain Conditions: A Systematic Review. Journal of Clinical Medicine vol. 11 Preprint at 10.3390/jcm11164813 (2022). 10.3390/jcm11164813 PMC9409905 36013053

[11] Santos J, Martins S, Azevedo LF, Fernandes L. Pain as a risk factor for suicidal behavior in older adults: A systematic review. Archives of Gerontology and Geriatrics vol. 87 Preprint at 10.1016/j.archger.2019.104000 (2020). 10.1016/j.archger.2019.104000 31891889

[12] Torino G, et al. Physical pain and suicide-related outcomes across the lifespan: systematic review and meta-analysis. Psychiatry Res. 2025;345:116371. 39889568 10.1016/j.psychres.2025.116371

[13] Verrocchio MC et al. Mental pain and suicide: A systematic review of the literature. Frontiers in Psychiatry vol. 7 Preprint at 10.3389/fpsyt.2016.00108 (2016). 10.3389/fpsyt.2016.00108 PMC4913233 27378956

[14] Wang T, Yang L, Liu B-P, Jia C-X. The relationship between psychological pain and suicidality in patients with major depressive disorder: A meta-analysis. J Affect Disord. 2024;346:115–21. 37926158 10.1016/j.jad.2023.10.160

[15] Tymofiyeva O et al. A Systematic Review of MRI Studies and the ‘Emotional paiN and social Disconnect (END)’ Brain Model of Suicidal Behavior in Youth. Behavioural Neurology vol. 2023 Preprint at 10.1155/2023/7254574 (2023). 10.1155/2023/7254574 PMC10541999 37786433

[16] Colino L et al. Cannabinoid Receptors, Mental Pain and Suicidal Behavior: a Systematic Review. Current Psychiatry Reports vol. 20 Preprint at 10.1007/s11920-018-0880-4 (2018). 10.1007/s11920-018-0880-4 29546501

[17] Ducasse D, et al. Psychological pain in suicidality: A Meta-Analysis. J Clin Psychiatry. 2018;79:16r10732. 28872267 10.4088/JCP.16r10732

[18] Klonsky ED, Pachkowski MC, Shahnaz A, May AM. The three-step theory of suicide: description, evidence, and some useful points of clarification. Prev Med (Baltim) 152, (2021). 10.1016/j.ypmed.2021.106549 34538372

[19] O’Connor RC, Kirtley OJ. The integrated motivational-volitional model of suicidal behaviour. Philosophical Transactions of the Royal Society B: Biological Sciences vol. 373 Preprint at 10.1098/rstb.2017.0268 (2018). 10.1098/rstb.2017.0268 PMC6053985 30012735

[20] Van Orden KA, et al. The interpersonal theory of suicide. Psychol Rev. 2010;117:575–600. 20438238 10.1037/a0018697 PMC3130348

[21] Risch N, et al. Pain tolerance and threshold in suicide attempters: A systematic review and meta-analysis. Psychiatry Res Vol. 2024. 10.1016/j.psychres.2023.115618. 331 Preprint at. 10.1016/j.psychres.2023.115618 38071878

[22] Shneidman E. Suicide as psychache: A clinical approach to Self-Destructive behavior. Jason Aronson; 1993.

[23] Dantzer R, O’Connor JC, Freund GG, Johnson RW, Kelley KW. From inflammation to sickness and depression: When the immune system subjugates the brain. Nature Reviews Neuroscience vol. 9 46–56 Preprint at 10.1038/nrn2297 (2008). 10.1038/nrn2297 PMC2919277 18073775

[24] Berntson GG, Khalsa SS. Neural Circuits of Interoception. Trends in Neurosciences vol. 44 17–28 Preprint at 10.1016/j.tins.2020.09.011 (2021). 10.1016/j.tins.2020.09.011 PMC8054704 33378653

[25] Marchand F, Perretti M, McMahon SB. Role of the immune system in chronic pain. Nature Reviews Neuroscience vol. 6 521–532 Preprint at 10.1038/nrn1700 (2005). 10.1038/nrn1700 15995723

[26] Chopra K, Arora V. An intricate relationship between pain and depression: Clinical correlates, coactivation factors and therapeutic targets. Expert Opinion on Therapeutic Targets vol. 18 159–176 Preprint at 10.1517/14728222.2014.855720 (2014). 10.1517/14728222.2014.855720 24295272

[27] Sluka KA, Clauw DJ. Neurobiology of fibromyalgia and chronic widespread pain. Neuroscience vol. 338 114–129 Preprint at 10.1016/j.neuroscience.2016.06.006 (2016). 10.1016/j.neuroscience.2016.06.006 PMC5083139 27291641

[28] Sommer C, Leinders M, Üçeyler N. Inflammation in the pathophysiology of neuropathic pain. Pain. 2018;159:595–602. 29447138 10.1097/j.pain.0000000000001122

[29] Edvinsson L, Haanes KA, Warfvinge K. Does inflammation have a role in migraine? Nature Reviews Neurology vol. 15 483–490 Preprint at 10.1038/s41582-019-0216-y (2019). 10.1038/s41582-019-0216-y 31263254

[30] Savitz J, Harrison NA. Interoception and Inflammation in Psychiatric Disorders. Biological Psychiatry: Cognitive Neuroscience and Neuroimaging vol. 3 514–524 Preprint at 10.1016/j.bpsc.2017.12.011 (2018). 10.1016/j.bpsc.2017.12.011 PMC5995132 29884282

[31] Benson S, et al. Amplified gut feelings under inflammation and depressed mood: A randomized fMRI trial on interoceptive pain in healthy volunteers. Brain Behav Immun. 2023;112:132–7. 37302437 10.1016/j.bbi.2023.06.005

[32] Benson S, et al. Neural circuitry mediating inflammation-induced central pain amplification in human experimental endotoxemia. Brain Behav Immun. 2015;48:222–31. 25882910 10.1016/j.bbi.2015.03.017

[33] Ji RR, Nackley A, Huh Y, Terrando N, Maixner W. Neuroinflammation and central sensitization in chronic and widespread pain. Anesthesiology. 2018;129:343–66. 29462012 10.1097/ALN.0000000000002130 PMC6051899

[34] Fang X-X et al. Inflammation in pathogenesis of chronic pain: foe and friend. Mol Pain 19, (2023). 10.1177/17448069231178176 PMC10214073 37220667

[35] Amiri M, Alavinia M, Singh M, Kumbhare D. Pressure Pain Threshold in Patients with Chronic Pain: A Systematic Review and Meta-Analysis. American Journal of Physical Medicine and Rehabilitation vol. 100 656–674 Preprint at 10.1097/PHM.0000000000001603 (2021). 10.1097/PHM.0000000000001603 33002911

[36] Irwin MR, Olmstead R, Bjurstrom MF, Finan PH, Smith MT. Sleep disruption and activation of cellular inflammation mediate heightened pain sensitivity: A randomized clinical trial. Pain. 2023;164:1128–37. 36314570 10.1097/j.pain.0000000000002811 PMC10106531

[37] Pawlik RJ, et al. Inflammation shapes neural processing of interoceptive fear predictors during extinction learning in healthy humans. Brain Behav Immun. 2023;108:328–39. 36535608 10.1016/j.bbi.2022.12.010

[38] Craig AD. How do you feel? Interoception: the sense of the physiological condition of the body. Nat Rev Neurosci. 2002;3:655–66. 12154366 10.1038/nrn894

[39] Craig AD. Interoception: the sense of the physiological condition of the body. Curr Opin Neurobiol. 2003;13:500–5. 12965300 10.1016/s0959-4388(03)00090-4

[40] Garfinkel SN, Seth AK, Barrett AB, Suzuki K, Critchley H. D. Knowing your own heart: distinguishing interoceptive accuracy from interoceptive awareness. Biol Psychol. 2015;104:65–74. 25451381 10.1016/j.biopsycho.2014.11.004

[41] Mehling WE, et al. The multidimensional assessment of interoceptive awareness (MAIA). PLoS ONE. 2012;7:e48230. 23133619 10.1371/journal.pone.0048230 PMC3486814

[42] Adolfi F, et al. Convergence of interoception, emotion, and social cognition: A twofold fMRI meta-analysis and lesion approach. Cortex. 2017;88:124–42. 28088652 10.1016/j.cortex.2016.12.019

[43] Yoris A et al. The roles of interoceptive sensitivity and metacognitive interoception in panic. Behav Brain Funct 11, (2015). 10.1186/s12993-015-0058-8 PMC4422149 25889157

[44] Petrini L, Arendt-Nielsen L. Understanding Pain Catastrophizing: Putting Pieces Together. Frontiers in Psychology vol. 11 Preprint at 10.3389/fpsyg.2020.603420 (2020). 10.3389/fpsyg.2020.603420 PMC7772183 33391121

[45] Jiang Y, et al. Perturbed connectivity of the amygdala and its subregions with the central executive and default mode networks in chronic pain. Pain. 2016;157:1970–8. 27168362 10.1097/j.pain.0000000000000606 PMC6647855

[46] Eisenberger NI. The pain of social disconnection: Examining the shared neural underpinnings of physical and social pain. Nature Reviews Neuroscience vol. 13 421–434 Preprint at 10.1038/nrn3231 (2012). 10.1038/nrn3231 22551663

[47] Meerwijk EL, Ford JM, Weiss SJ. Brain regions associated with psychological pain: implications for a neural network and its relationship to physical pain. Brain Imaging Behav. 2013;7:1–14. 22660945 10.1007/s11682-012-9179-y

[48] Apkarian AV, Bushnell MC, Treede RD, Zubieta JK. Human brain mechanisms of pain perception and regulation in health and disease. Eur J Pain. 2005;9:463. 15979027 10.1016/j.ejpain.2004.11.001

[49] Mee S, Bunney BG, Reist C, Potkin SG, Bunney WE. Psychological pain: A review of evidence. Journal of Psychiatric Research vol. 40 680–690 Preprint at 10.1016/j.jpsychires.2006.03.003 (2006). 10.1016/j.jpsychires.2006.03.003 16725157

[50] Courtet P, Guillaume S. Learning from Artemisia’s lucretia: embodied suffering and interoception in suicide. Front Psychiatry 11, (2020). 10.3389/fpsyt.2020.00758 PMC7412131 32848933

[51] Oquendo MA, et al. Toward a biosignature for suicide. Am J Psychiatry. 2014;171:1259–77. 25263730 10.1176/appi.ajp.2014.14020194 PMC4356635

[52] Courtet P et al. Neuroinflammation in suicide: Toward a comprehensive model. World Journal of Biological Psychiatry vol. 17 564–586 Preprint at 10.3109/15622975.2015.1054879 (2016). 10.3109/15622975.2015.1054879 26223957

[53] Bengoechea-Fortes S, de la Ramírez-Expósito P, M. J., Martínez-Martos JM. Suicide, neuroinflammation and other physiological alterations. European Archives of Psychiatry and Clinical Neuroscience vol. 274 1037–1049 Preprint at 10.1007/s00406-023-01584-z (2024). 10.1007/s00406-023-01584-z PMC10009854 36913003

[54] Olié E et al. History of suicidal behaviour and analgesic use in community-dwelling elderly. Psychotherapy and Psychosomatics vol. 82 341–343 Preprint at 10.1159/000350504 (2013). 10.1159/000350504 23942382

[55] Calati R, Olié E, Ritchie K, Artero S, Courtet P. Suicidal Ideation and Suicide Attempts in the Elderly Associated with Opioid Use and Pain Sensitivity. Psychotherapy and Psychosomatics vol. 86 373–375 Preprint at 10.1159/000478021 (2017). 10.1159/000478021 29131065

[56] Hielscher E, Zopf R. Interoceptive abnormalities and suicidality: A systematic review. Behav Ther. 2021;52:1035–54. 34452660 10.1016/j.beth.2021.02.012

[57] Smith AR, Duffy ME, Joiner TE. Introduction to the special issue on interoception and suicidality. Behav Ther. 2021;52:1031–4. 34452659 10.1016/j.beth.2021.06.003

[58] Rogers ML, Hagan CR, Joiner TE. Examination of interoception along the suicidality continuum. J Clin Psychol. 2018;74:1004–16. 29319196 10.1002/jclp.22564

[59] Gioia AN, Forrest LN, Smith AR. Diminished body trust uniquely predicts suicidal ideation and nonsuicidal self-injury among people with recent self-injurious thoughts and behaviors. Suicide Life Threat Behav. 2022;52:1205–16. 36029117 10.1111/sltb.12915

[60] Elman I, Borsook D, Volkow ND. Pain and suicidality: Insights from reward and addiction neuroscience. Progress in Neurobiology vol. 109 1–27 Preprint at 10.1016/j.pneurobio.2013.06.003 (2013). 10.1016/j.pneurobio.2013.06.003 PMC4827340 23827972

[61] Orbach I. Dissociation, physical pain, and suicide: A hypothesis. Suicide Life Threat Behav. 1994;24:68–79. 8203010

[62] Orbach I, Mikulincer M, King R, Cohen D, Stein D. Thresholds and tolerance of physical pain in suicidal and nonsuicidal adolescents. J Consult Clin Psychol. 1997;65:646–52. 9256566 10.1037//0022-006x.65.4.646

[63] Orbach I, et al. Perception of physical pain in accident and suicide attempt patients: Self-preservation vs self-destruction. J Psychiatr Res. 1996;30:307. 8905539 10.1016/0022-3956(96)00008-8

[64] Orbach I, et al. Tolerance for physical pain in suicidal subjects. Death Stud. 1996;20:327–41. 10160569 10.1080/07481189608252786

[65] Orbach I, Stein D, Shani-Sela M, Har‐Even D. Body attitudes and body experiences in suicidal adolescents. Suicide Life Threat Behav. 2001;31:237–49. 11577910 10.1521/suli.31.3.237.24250

[66] Orbach I, Lotem-Peleg M, Kedem P. Attitudes toward the body in suicidal, depressed, and normal adolescents. Suicide Life Threat Behav. 1995;25:211–21. 7570782

[67] Joiner TE. Why people die by suicide. Cambridge: Harvard University Press; 2005.

[68] Paashaus L, et al. Do suicide attempters and suicide ideators differ in capability for suicide? Psychiatry Res. 2019;275:304–9. 30953875 10.1016/j.psychres.2019.03.038

[69] Cáceda R, Kordsmeier NC, Golden E, Gibbs HM, Delgado PL. Differential processing of physical and psychological pain during acute suicidality. Psychotherapy and Psychosomatics vol. 86 116–118 Preprint at 10.1159/000450713 (2017). 10.1159/000450713 28183088

[70] Ludäscher P, et al. Pain sensitivity and neural processing during dissociative States in patients with borderline personality disorder with and without comorbid posttraumatic stress disorder: A pilot study. J Psychiatry Neurosci. 2010;35:177–84. 20420768 10.1503/jpn.090022 PMC2861134

[71] Röder CH, Michal M, Overbeck G, Van De Ven VG, Linden DE. J. Pain response in depersonalization: A functional imaging study using hypnosis in healthy subjects. Psychother Psychosom. 2007;76:115–21. 17230052 10.1159/000097970

[72] Rousseaux F et al. Lippincott Williams and Wilkins,. Virtual reality and hypnosis for anxiety and pain management in intensive care units: A prospective randomised trial among cardiac surgery patients. in European Journal of Anaesthesiology vol. 39 58–66 (2022). 10.1097/EJA.0000000000001633 PMC8654253 34783683

[73] Calati R, Bensassi I, Courtet P. The link between dissociation and both suicide attempts and non-suicidal self-injury: Meta-analyses. Psychiatry Research vol. 251 103–114 Preprint at 10.1016/j.psychres.2017.01.035 (2017). 10.1016/j.psychres.2017.01.035 28196773

[74] Foote B, Smolin Y, Neft DI, Lipschitz D. Dissociative disorders and suicidality in psychiatric outpatients. J Nerv Mental Disease. 2008;196:29–36. 10.1097/NMD.0b013e31815fa4e7 18195639

[75] Saxe GN, Chawla N, Van der Kolk B. Self-Destructive behavior in patients with dissociative disorders. Suicide Life Threat Behav. 2002;32:313–20. 12374476 10.1521/suli.32.3.313.22174

[76] Schäflein E, Sattel HC, Pollatos O, Sack M. Disconnected - impaired interoceptive accuracy and its association with self-perception and cardiac vagal tone in patients with dissociative disorder. Front Psychol 9, (2018). 10.3389/fpsyg.2018.00897 PMC6031288 29997537

[77] Ducasse D, Courtet P, Olié E. Physical and social pains in borderline disorder and neuroanatomical correlates: A systematic review. Current Psychiatry Reports vol. 16 Preprint at 10.1007/s11920-014-0443-2 (2014). 10.1007/s11920-014-0443-2 24633938

[78] Pavony MT, Lenzenweger MF. Somatosensory processing and borderline personality disorder features: a signal detection analysis of proprioception and exteroceptive sensitivity. J Pers Disord. 2013;27:208–21. 23514184 10.1521/pedi.2013.27.2.208

[79] Schmahl C, Bremner JD. Neuroimaging in borderline personality disorder. J Psychiatr Res. 2006;40:419–27. 16239012 10.1016/j.jpsychires.2005.08.011 PMC3233768

[80] Niedtfeld I et al. Functional connectivity of pain-mediated affect regulation in borderline personality disorder. PLoS ONE 7, (2012). 10.1371/journal.pone.0033293 PMC3299768 22428013

[81] Craig AD. B. The sentient self. Brain structure & function vol. 214 563–577 Preprint at 10.1007/s00429-010-0248-y (2010). 10.1007/s00429-010-0248-y 20512381

[82] Allen M. Unravelling the Neurobiology of Interoceptive Inference. Trends in Cognitive Sciences vol. 24 265–266 Preprint at 10.1016/j.tics.2020.02.002 (2020). 10.1016/j.tics.2020.02.002 32160563

[83] Gélébart J, Garcia-Larrea L, Frot M. Amygdala and anterior Insula control the passage from nociception to pain. Cereb Cortex. 2023;33:3538–47. 35965070 10.1093/cercor/bhac290

[84] Pollatos O, Füstös J, Critchley HD. On the generalised embodiment of pain: how interoceptive sensitivity modulates cutaneous pain perception. Pain. 2012;153:1680–6. 22658270 10.1016/j.pain.2012.04.030

[85] Salomons TV, Johnstone T, Backonja M-M, Shackman AJ, Davidson RJ. Individual differences in the effects of perceived controllability on pain perception: critical role of the prefrontal cortex. J Cogn Neurosci. 2007;19:993–1003. 17536969 10.1162/jocn.2007.19.6.993

[86] Lorenz J, Minoshima S, Casey KL. Keeping pain out of Mind: the role of the dorsolateral prefrontal cortex in pain modulation. Brain. 2003;126:1079–91. 12690048 10.1093/brain/awg102

[87] Etkin A, Prater KE, Hoeft F, Menon V, Schatzberg A. Failure of anterior cingulate activation and connectivity with the amygdala during implicit regulation of emotional processing in generalized anxiety disorder. Am J Psychiatry. 2010;167:545–54. 20123913 10.1176/appi.ajp.2009.09070931 PMC4367202

[88] Xiao X, Zhang YQ. A new perspective on the anterior cingulate cortex and affective pain. Neuroscience and Biobehavioral Reviews vol. 90 200–211 Preprint at 10.1016/j.neubiorev.2018.03.022 (2018). 10.1016/j.neubiorev.2018.03.022 29698697

[89] Ong WY, Stohler CS, Herr DR. Role of the Prefrontal Cortex in Pain Processing. Molecular Neurobiology vol. 56 1137–1166 Preprint at 10.1007/s12035-018-1130-9 (2019). 10.1007/s12035-018-1130-9 PMC6400876 29876878

[90] Gamal-Eltrabily M, Martínez-Lorenzana G, González-Hernández A, Condés-Lara M. Cortical Modulation of Nociception. Neuroscience vol. 458 256–270 Preprint at 10.1016/j.neuroscience.2021.01.001 (2021). 10.1016/j.neuroscience.2021.01.001 33465410

[91] Lanius RA et al. Emotion modulation in PTSD: Clinical and neurobiological evidence for a dissociative subtype. American Journal of Psychiatry vol. 167 640–647 Preprint at 10.1176/appi.ajp.2009.09081168 (2010). 10.1176/appi.ajp.2009.09081168 PMC3226703 20360318

[92] Roydeva MI, Reinders AATS. Biomarkers of Pathological Dissociation: A Systematic Review. Neuroscience and Biobehavioral Reviews vol. 123 120–202 Preprint at 10.1016/j.neubiorev.2020.11.019 (2021). 10.1016/j.neubiorev.2020.11.019 33271160

[93] Cavicchioli M, et al. Neural responses to emotional stimuli across the dissociative spectrum: common and specific mechanisms PCN psychiatry and clinical neurosciences. Psychiatry Clin Neurosci. 2023;77:315–29. 36938718 10.1111/pcn.13547

[94] Wang S, et al. Resting-state neural mechanisms of capability for suicide and their interaction with pain – A CAN-BIND-05 study. J Affect Disord. 2023;330:139–47. 36878406 10.1016/j.jad.2023.02.147

[95] Lanius RA, et al. A review of the Neurobiological basis of Trauma-Related dissociation and its relation to Cannabinoid- and Opioid-Mediated stress response: a transdiagnostic, translational approach. Curr Psychiatry Rep. 2018;20:118. 30402683 10.1007/s11920-018-0983-y

[96] Spiegel D et al. Dissociative disorders in DSM-5. Annual Review of Clinical Psychology vol. 9 299–326 Preprint at 10.1146/annurev-clinpsy-050212-185531 (2013). 10.1146/annurev-clinpsy-050212-185531 23394228

[97] American Psychiatric Association. Diagnostic and statistical manual of mental disorders. American Psychiatric Association Publishing; 2022. 10.1176/appi.books.9780890425787.

[98] Lanius RA, Brand B, Vermetten E, Frewen PA, Spiegel D. The dissociative subtype of posttraumatic stress disorder: Rationale, clinical and neurobiological evidence, and implications. Depression and Anxiety vol. 29 701–708 Preprint at 10.1002/da.21889 (2012). 10.1002/da.21889 22431063

[99] Perry BD, Pollard RA, Blakley L, Baker WL, Vigilante D. Childhood trauma, the neurobiology of adaptation, and ‘Use-Dependent’ development of the brain: how ‘States’ become ‘traits’. Infant Mental Health J 16 (1995).

[100] Bremner DJ. Acute and chronic responses to psychological trauma: where do we go from here?? Am J Psychiatry. 1999;156:349–500. 10080546 10.1176/ajp.156.3.349

[101] Schore AN. The effects of early relational trauma on right brain development, affect regulation, and infant mental health. Infant Ment Health J. 2001;22:201–69.

[102] Schore AN. Dysregulation of the right brain: a fundamental mechanism of traumatic attachment and the psychopathogenesis of posttraumatic stress disorder. Australian New Z J Psychiatry. 2002;36:9–30. 11929435 10.1046/j.1440-1614.2002.00996.x

[103] Schore AN. Relational trauma and the developing right brain: an interface of psychoanalytic self psychology and neuroscience. Ann N Y Acad Sci. 2009;1159:189–203. 19379241 10.1111/j.1749-6632.2009.04474.x

[104] Schore AN. Relational trauma, brain development, and dissociation. In: Ford JD, Courtois CA, editors. Treating complex traumatic stress disorders in children and adolescents: scientific foundations and therapeutic models. New York: Guildford; 2013. pp. 3–23.

[105] Rogier G, et al. The multifaceted role of emotion regulation in suicidality: systematic reviews and Meta-Analytic evidence. Psychol Bull. 2024;150:45–81. 38376911 10.1037/bul0000415

[106] Antoniou G, Lambourg E, Steele JD, Colvin LA. The effect of adverse childhood experiences on chronic pain and major depression in adulthood: a systematic review and meta-analysis. British Journal of Anaesthesia vol. 130 729–746 Preprint at 10.1016/j.bja.2023.03.008 (2023). 10.1016/j.bja.2023.03.008 PMC10251130 37087334

[107] Dalechek DE, Caes L, McIntosh G, Whittaker AC. Anxiety, history of childhood adversity, and experiencing chronic pain in adulthood: A systematic literature review and meta-analysis. Eur J Pain (United Kingdom). 2024;28:867–85. 10.1002/ejp.2232 38189218

[108] Vonderlin R, et al. Dissociation in victims of childhood abuse or neglect: A meta-Analytic review. Psychol Med. 2018;48:2467–76. 29631646 10.1017/S0033291718000740

[109] Lebois LAM, et al. Large-scale functional brain network architecture changes associated with trauma-related dissociation. Am J Psychiatry. 2021;178:165–73. 32972201 10.1176/appi.ajp.2020.19060647 PMC8030225

[110] Lebois LAM, et al. Deconstructing dissociation: a triple network model of trauma-related dissociation and its subtypes. Neuropsychopharmacology. 2022;47:2261–70. 36202907 10.1038/s41386-022-01468-1 PMC9630268

[111] Reisch T, et al. An fMRI study on mental pain and suicidal behavior. J Affect Disord. 2010;126:321–5. 20434779 10.1016/j.jad.2010.03.005

[112] Ernst J, et al. The association of interoceptive awareness and alexithymia with neurotransmitter concentrations in Insula and anterior cingulate. Soc Cogn Affect Neurosci. 2013;9:857–63. 23596189 10.1093/scan/nst058 PMC4040102

[113] Bernstein EM, Putnam FW. Development, reliability, and validity of a dissociation scale. J Nerv Ment Dis. 1986;174:727–35. 3783140 10.1097/00005053-198612000-00004

[114] Butler LD, Normative, Dissociation. Psychiatr Clin North Am. 2006;29:45–62. 16530586 10.1016/j.psc.2005.10.004

[115] Giesbrecht T, Lynn SJ, Lilienfeld SO, Merckelbach H. Cognitive processes in dissociation: an analysis of core theoretical assumptions. Psychol Bull. 2008;134:617–47. 18729565 10.1037/0033-2909.134.5.617

[116] Badura Brack AS et al. Neurostructural brain imaging study of trait dissociation in healthy children. BJPsych Open 8, (2022). 10.1192/bjo.2022.576 PMC9534905 36148845

[117] Dorahy MJ, Gold SN, O’Neil JA. Dissociation and the dissociative disorders. New York: Routledge; 2022. 10.4324/9781003057314.

[118] Dell PF, O’Neil JA. Dissociation and the dissociative disorders: DSM-V and beyond. Routledge; 2010.

[119] Nijenhuis ERS. Somatoform dissociation: major symptoms of dissociative disorders. J Trauma Dissociation. 2001;1:7–32.

[120] Nijenhuis ERS, van der Hart O. In: Goodwin J, Attias R, editors. Forgetting and reexperiencing trauma: from anesthesia to pain. In splintered reflections: images of the body In trauma. Basic Books/Hachette Book Group; 1999. pp. 39–65.

[121] Carlson EB, Putnam FW. An update on the dissociative experiences scale. Dissociation: Progress Dissociative Disorders. 1993;6:16–27.

[122] Leonard KN, Telch MJ, Harrington PJ. Dissociation in the laboratory: a comparison of strategies. Behav Res Ther. 1999. 10.1016/S0005-7967(98)00072-2. 9922557 10.1016/s0005-7967(98)00072-2

[123] Levinger S, Somer E, Holden RR. The importance of mental pain and physical dissociation in youth suicidality. J Trauma Dissociation. 2015;16:322–39. 25760400 10.1080/15299732.2014.989644

[124] Bernanke JA, Stanley BH, Oquendo MA. Toward fine-grained phenotyping of suicidal behavior: The role of suicidal subtypes. Molecular Psychiatry vol. 22 1080–1081 Preprint at 10.1038/mp.2017.123 (2017). 10.1038/mp.2017.123 PMC5785781 28607457

[125] Kim H, et al. Classification of attempted suicide by cluster analysis: A study of 888 suicide attempters presenting to the emergency department. J Affect Disord. 2018;235:184–90. 29656265 10.1016/j.jad.2018.04.001

[126] Lopez-Castroman J, Nogue E, Guillaume S, Picot MC, Courtet P. Clustering suicide attempters. J Clin Psychiatry. 2016;77:e711–8. 27035768 10.4088/JCP.15m09882

[127] Bryan CJ et al. Nonlinear change processes and the emergence of suicidal behavior: A conceptual model based on the fluid vulnerability theory of suicide. New Ideas Psychol 57, (2020). 10.1016/j.newideapsych.2019.100758 PMC7050543 32123464

[128] Dutra L, Bureau JF, Holmes B, Lyubchik A, Lyons-Ruth K. Quality of early care and childhood trauma: A prospective study of developmental pathways to dissociation. J Nerv Mental Disease. 2009;197:383–90. 10.1097/NMD.0b013e3181a653b7 PMC2697443 19525736

[129] Quiñones MA. Adverse childhood experiences and dissociative disorders: a causal pathway based on the disruptive impacts of cumulative childhood adversity and distress-related dissociation. in Dissociation and Dissociative Disorders: Past, Present, Future (eds. Dorahy, M. J., Gold, S. N. & O’Neil, J.) 209–222 (Taylor & Francis, 2022).

[130] Kate M-A, Jamieson G, Middleton W. Childhood sexual, emotional, and physical abuse as predictors of dissociation in adulthood. J Child Sex Abus. 2021;30:953–76. 34353238 10.1080/10538712.2021.1955789

[131] Hochschild A, Grunebaum MF, Mann JJ. The rapid anti-suicidal ideation effect of ketamine: A systematic review. Prev Med (Baltim) 152, (2021). 10.1016/j.ypmed.2021.106524 34538369

[132] Reinstatler L, Youssef NA. Ketamine as a Potential Treatment for Suicidal Ideation: A Systematic Review of the Literature. Drugs in R and D vol. 15 37–43 Preprint at 10.1007/s40268-015-0081-0 (2015). 10.1007/s40268-015-0081-0 PMC4359177 25773961

[133] Siegel AN et al. Antisuicidal and antidepressant effects of ketamine and esketamine in patients with baseline suicidality: A systematic review. *Journal of Psychiatric Research* vol. 137 426–436 Preprint at 10.1016/j.jpsychires.2021.03.009 (2021). 10.1016/j.jpsychires.2021.03.009 33774537

[134] Muscat SA, Hartelius G, Crouch CR, Morin KW. An integrative approach to ketamine therapy May enhance multiple dimensions of efficacy: improving therapeutic outcomes with treatment resistant depression. Front Psychiatry 12, (2021). 10.3389/fpsyt.2021.710338 PMC8653702 34899408

[135] Morena M et al. Ketamine anesthesia enhances fear memory consolidation via noradrenergic activation in the basolateral amygdala. Neurobiol Learn Mem 178, (2021). 10.1016/j.nlm.2020.107362 33333316

[136] Wolfson P, Vaid G. Ketamine-assisted psychotherapy, psychedelic methodologies, and the impregnable value of the subjective—a new and evolving approach. Front Psychiatry 15, (2024). 10.3389/fpsyt.2024.1209419 PMC10867319 38362026

